# Stable Formulations of Peptide-Based Nanogels

**DOI:** 10.3390/molecules25153455

**Published:** 2020-07-29

**Authors:** Elisabetta Rosa, Carlo Diaferia, Enrico Gallo, Giancarlo Morelli, Antonella Accardo

**Affiliations:** 1 Department of Pharmacy and Interuniversity Research Centre on Bioactive Peptides (CIRPeB), University of Naples “Federico II”, via Mezzocannone 16, 80134 Naples, Italy; elisabetta.rosa@unina.it (E.R.); carlo.diaferia@unina.it (C.D.); gmorelli@unina.it (G.M.); 2 IRCCS SDN, Via E. Gianturco 113, 80143 Naples, Italy; enrico.gallo@synlab.it

**Keywords:** peptide-based nanogels, inverse emulsion, nanogel formulation, hydrogel nanoparticles, diagnostic imaging, peptide aggregates, doxorubicin

## Abstract

Recently, nanogels have been identified as innovative formulations for enlarging the application of hydrogels (HGs) in the area of drug delivery or in diagnostic imaging. Nanogels are HGs-based aggregates with sizes in the range of nanometers and formulated in order to obtain injectable preparations. Regardless of the advantages offered by peptides in a hydrogel preparation, until now, only a few examples of peptide-based nanogels (PBNs) have been developed. Here, we describe the preparation of stable PBNs based on Fmoc-Phe-Phe-OH using three different methods, namely water/oil emulsion (W/O), top-down, and nanogelling in water. The effect of the hydrophilic–lipophilic balance (HLB) in the formulation was also evaluated in terms of size and stability. The resulting nanogels were found to encapsulate the anticancer drug doxorubicin, chosen as the model drug, with a drug loading comparable with those of the liposomes.

## 1. Introduction

**Figure 1:**
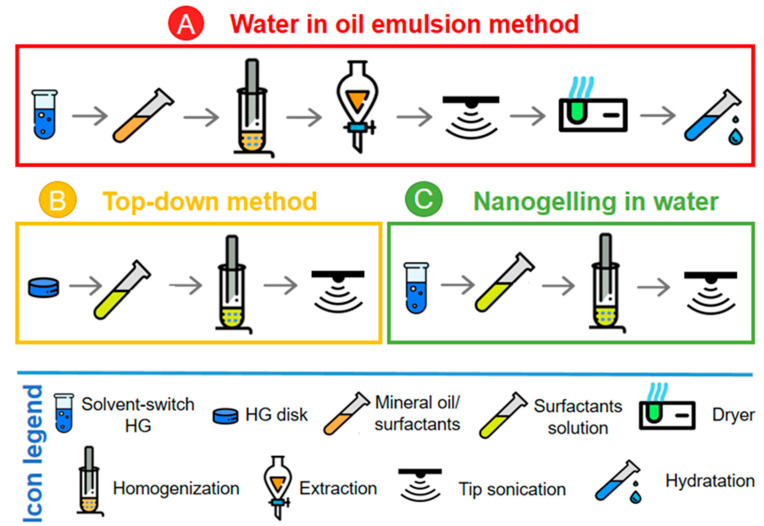
Icons graphical representation of the three different strategies for the nanogel formulation. (**A**) Water-in-oil emulsion methodology; (**B**) Top-down method; and (**C**) Nanogelling in water approach. Each step is identified using an icon of which the legend is reported too.

In the last few years, the design and the formulation of hydrogel materials (HGs) have attracted a great deal of interest in biomedical areas [1,2]. Hydrogels are three-dimensional (3D) structures formed by chemically or physically crosslinked polymeric networks which can confine a large amount of water. Because of their unique structural and mechanical properties and their high biocompatibility, HGs have been proposed as innovative materials for different biomedical applications such as tissue engineering [3,4,5,6] and delivery systems of active pharmaceutical ingredients (APIs), including drugs [7] or diagnostic agents [8]. Nanosized structures, including micelles [9], nanoparticles [10], nanofibers [11], and liposomes [12] are particularly suitable for this purpose. Indeed, they overcome some problematic issues and limitations related to the in vivo bioavailability, biodisponibility, toxicity, and instability of drugs. Hydrogel nanoparticles, also named nanogels, seem to be particularly appealing for this purpose. They combine the advantages of traditional HGs and nanosized particles. Nanogels are aggregates in the submicron range scale composed of an interior hydrogel-like network (core) stabilized by an external surfactant coating (shell). Their physical colloidal form is compatible with needle injection. The large surface area is accessible for multivalent bioconjugation, meanwhile, the interior network can work as a reservoir for additional incorporation of biomolecules. Moreover, the small size of these formulations generally leads to simple renal clearance, increased penetration through tissue barriers, and good stability for prolonged circulation in the blood stream. Analogously to HGs, nanogels can be prepared by the self-assembly of biocompatible and biodegradable polymers such as chitosan (CS) [13], hyaluronic acid (HA) [14], and dextran [15] according to different technological approaches including microfluidic [16] micromolding [17] and photolithographic techniques [18]. However, most of these processes require extreme conditions of pH and temperature and some of them require the use of crosslinking agents, which are not completely biocompatible. In order to overcome these limitations, peptide-based nanogels (PBNs) could represent an innovative tool. In the literature, so far, there are only two examples of peptide-based nanogels. The first example describes the preparation of the nanogels by the inverse emulsion technique [19] and the other example describes a top-down methodology. On the one hand, the inverse emulsion technique consists of the formation of a water-in-oil (W/O) emulsion via homogenization of an aqueous phase containing the Fmoc-Phe-Phe-OH (Fmoc-FF, *N*^α^-9-fluorenylmethoxycarbonyl-diphenylalanine) peptide with an oily phase containing a surfactant as a stabilizer. This methodology is very often used for the formulation of polymeric nanogels [20]. On the other hand, the top-down methodology involves the formulation of nanogel particles using a α,β-dehydrophenylalanine (ΔPhe) containing dipeptide, H-Phe-ΔPhe-OH (phenylalanine-α,β-dehydrophenylalanine) [21]. Successively, preformed H-Phe-ΔPhe-OH gels are sonicated with a microprobe, obtaining a formulation with an average hydrodynamic radius (r_H_) of 150 nm. However, in this formulation a certain degree of particle instability, probably due to the absence of surfactant protective shell, was noticed with an increase of the radius up to 350 nm, after 60 h. By starting from the awareness that in vitro and in vivo stability represents an essential requirement for the development of biomedical nanosystems, we exploited three different methodologies (see Figure 1) to achieve a stable and reproducible formulation of peptide-based nanogels. The peptide chosen for nanogel preparation is the ultrashort Fmoc-FF peptide. It is well known that many short and ultrashort peptide sequences can spontaneously self-assemble into HGs, as a consequence of physical and noncovalent interactions, thus, avoiding the employment of crosslinking agents [22,23,24]. Among these peptides, one of the most studied is Fmoc-FF. It was identified simultaneously by Ulijn’s [25] and Gazit’s groups [26], in 2006. This simple building block, utilizing a β-sheet motif, efficiently assembles into nanostructured fibrous hydrogels under physiological conditions. Structural studies have been carried out on this dipeptide and some of its analogues, resulting in an extensive comprehension of the self-assembling mechanism [27,28,29,30]. However, it was observed that the mechanical properties of the resulting gel were deeply affected by the preparation method (pH and the solvent switch method [25,26]) and by the final solution conditions (pH and salt content) [31]. Because of a series of interesting features (such as a certain stability degree across a broad range of pH and temperatures, the presence of hollow cavities in the supramolecular architecture, and mechanical rigidity), Fmoc-FF hydrogel was also proposed as a potential scaffold material for tissue engineering or as a drug delivery system [5,26]. The methods we investigated for preparation of the Fmoc-FF-based nanogels are as follows: (A) the water-in-oil emulsion technique (or reverse emulsion technique, W/O); (B) the top-down methodology; and (C) a novel modified procedure designed by us and named “nanogelling in water”. In the inverse emulsion method, we focused our attention on the stabilizing capacity of the surfactant chosen for the nanogel preparation, basing our reasoning on the hydrophilic–lipophilic balance (HLB). This parameter, proposed by Griffin between the late 1940s and early 1950s [32,33] allows one to predict the stability of an aqueous phase in the oily phase, or vice versa. A comparison of results obtained using these three different methods in terms of size, stability, and feasibility, is reported. Moreover, the capability of the resulting PBNs to encapsulate drugs was checked by using doxorubicin as the model drug and the loading capability, in terms of drug loading content, was compared to that of the liposomal doxorubicin formulations currently in clinical use.

## 2. Results

### 2.1. Nanogel Formulation Methodologies

**Figure 2:**
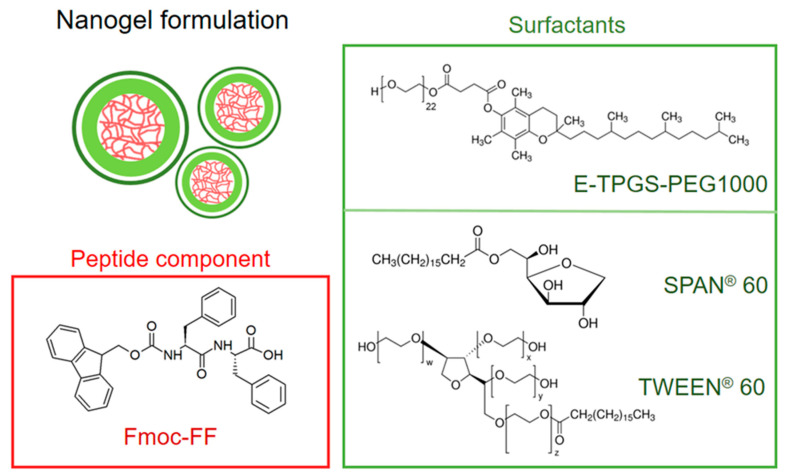
Chemical formulas of components used for the peptide-based nanogel formulation. Internal core (red) is formed by Fmoc-Phe-Phe-OH (Fmoc-FF). External surfactants shell (green) was formulated using d-α-tocopherol polyethylene glycol 1000 succinate (E-TPGS-PEG1000) or mixing SPAN^®^ 60 (sorbitan monostearate) and TWEEN^®^ 60 (polyethylene glycol sorbitan monostearate) in different ratios.

PBNs were prepared using Fmoc-FF peptide (Figure 2) which is well-known for its capability to self-assemble into macroscopic HGs [22,23]. Fmoc-FF-based nanogels were formulated according to the following three different preparation procedures (Figure 1): (A) water-in-oil inverse emulsion technique (W/O emulsion), (B) top-down approach, and (C) nanogelling in water. The inverse emulsion technique consists of the formation of a water-in-oil emulsion assisted by a mechanical homogenization step. The emulsion is achieved between an aqueous phase containing the gelling DMSO/H_2_O peptide solution and an organic phase consisting of a mineral oil, in which one or more stabilizing surfactants are dispersed. This method was previously proposed by Gazit’s group for fabrication of Fmoc-FF hydrogel nanoparticles coated using d-α-tocopheryl polyethylene glycol 1000 succinate (E-TPGS, Figure 2) surfactant as the stabilizing agent [24]. According to the reported experimental protocol, we prepared six nanogel formulations (see Table 1), all of them containing the same amount of Fmoc-FF (10 mg/mL, final gel at 0.25 %wt) and a different percentage of two biocompatible surfactants, TWEEN^®^ 60 (polyethylene glycol sorbitan monostearate, see Figure 2) and SPAN^®^ 60 (sorbitan stearate, see Figure 2). The combination of this couple of surfactants allows obtaining different HLB values (4.7 < HLB < 14.9) as indicated in Table 2. The HLB is equal to the molecular weight (MW) of the hydrophilic part of the surfactant divided by the molecular weight of the lipophilic part, multiplied by 20. It is a dimensionless quantity that can take values between 0 and 20.

$$HLB=\frac{MW\;hydrophilic\;part}{MW\;lipophilc\;part}\times 20$$

This approach permitted us to study the relationship between the HLB value and the structural properties of each formulation in terms of size, stability, and feasibility. The total amount of TWEEN^®^ 60 and SPAN^®^ 60 was 3 × 10^−5^ mol in all the nanogels, with respect to 1.86 × 10^−5^ mol of the Fmoc-FF (10 mg/mL). This ratio was chosen in order to assure a complete coating of the peptide core in the resulting nanoparticles. From the experimental point of view, 1 mL of the Fmoc-FF hydrogel, during its opaque-to-limpid transition step, was transferred into the mineral oil containing the two surfactants. The resulting suspension was mechanically homogenized for 5 min (at 35,000 rpm), and then the oily phase was extracted with n-hexane.

We also explored the possibility of preparing the PBNs according to a modified version of the “top-down” methodology, previously described by Chauhan et al. [23]. Their method for preparing Phe-∆Phe-based nanogels consisted of the progressive reduction in size of preformed hydrogels into an aqueous solution. In our approach, Fmoc-FF hydrogels were produced in macroscopic discs using silicone molds, and then they were unpacked into an aqueous solution of surfactants (TWEEN^®^/SPAN^®^ at 58/42 ratio, HLB = 10), using in sequence the homogenizer and the tip sonicator. Analogously to the W/O emulsion, also in this procedure, the amount of Fmoc-FF and of the surfactants were 10 mg/mL and 3 × 10^−5^ mol, respectively.

Beyond these two methods previously reported in the literature, nanogels were also formulated by a third procedure, which we named “nanogelling in water”, in which the peptide hydrogel was added to an aqueous solution of the stabilizing agents before the gelling procedure was completed. This methodology is very similar to the W/O emulsion method, but it avoids the use of mineral oil and, as a consequence, the use of n-hexane during the extraction step.

### 2.2. Nanogel Characterization

**Figure 3:**
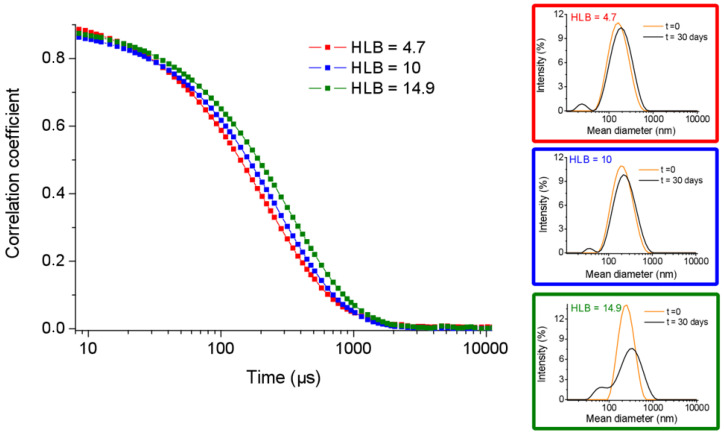
Intensity correlation functions for Fmoc-FF nanogels prepared according to the inverse emulsion at three different HLB values (4.7, 10, and 14.9). On the right, the dynamic light scattering (DLS) profiles for these formulations freshly prepared and after one month.

The dimensions of PBNs prepared according the previously procedures at different HLB values and their stability over the time were assessed by dynamic light scattering (DLS). Structural data (mean diameter, polydispersity index, PDI, and zeta potential) for all the samples are collected in Table 1. In Figure 3 are reported the intensity correlation functions for three HLB values (4.7, 10, and 14.9) that represent the two extreme HLB conditions and the middle condition.

**Figure 4:**
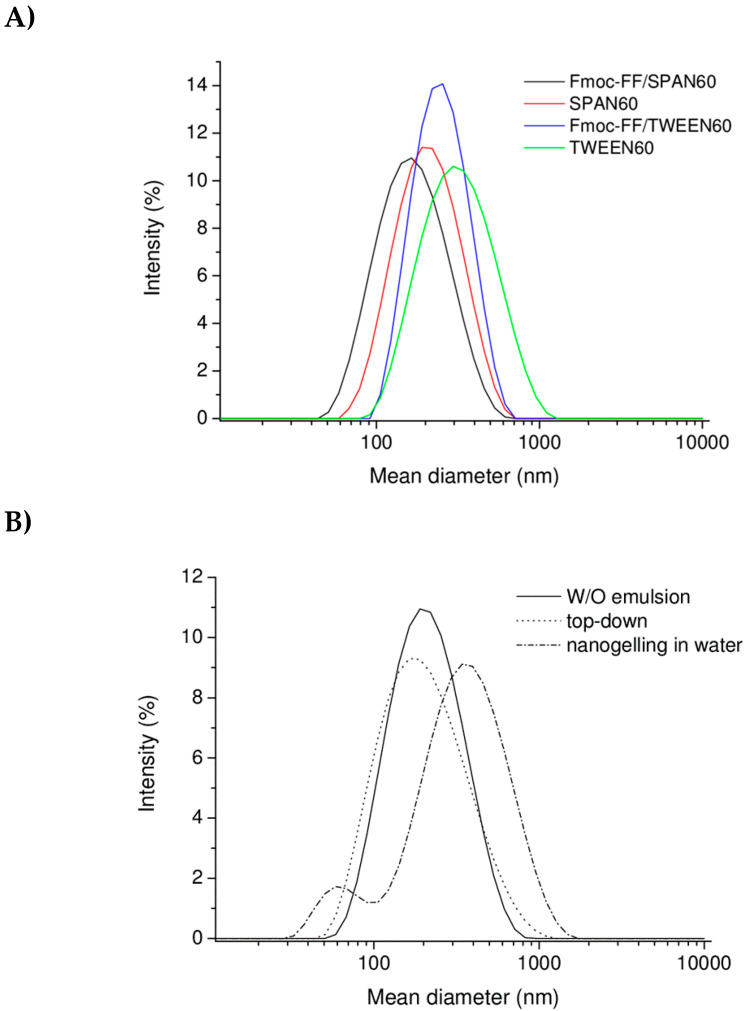
DLS profiles. (**A**) For Fmoc-FF/SPAN^®^60 (HLB = 4.7) and Fmoc-FF/TWEEN^®^60 (HLB = 14.9) as compared with the corresponding aggregates lacking the dipeptide; (**B**) For Fmoc-FF nanogels prepared according to W/O emulsion, top-down, and nanogelling in water methods (HLB = 10).

The DLS intensity profile of the three freshly prepared formulations were reported, as well as reported after one month. From the comparison of the mean diameter values (ranged between ~160 and ~240 nm) in Table 1 and of the intensity correlation functions in Figure 3, it seems that there is a direct correlation between the HLB value and the size of nanogels. This consideration is fully supported by the DLS measurements carried out on pure SPAN^®^ 60 and TWEEN^®^ 60 aggregates prepared with the same procedure without Fmoc-FF. Indeed, pure SPAN^®^ 60 and TWEEN^®^ 60 aggregates exhibit a hydrodynamic radius of 205 ± 111 and 310 ± 158 nm, respectively (see Figure 4A). These values are slightly higher (~20%) with respect to the values measured for the corresponding Fmoc-FF nanogels, thus, indicating that attractive interactions occur between the hydrophobic portion of the surfactants and the Fmoc-FF peptide.

Moreover, all the formulations, independent of the HLB value, present a shelf stability at room temperature up to a month, even if they showed slightly more uneven profiles at DLS analysis. For example, nanogels with an HLB value of 10 increased their size from 204 to 224 nm (around 10% with respect to the initial size). A similar behavior was also observed for the other formulations.

This result can be explained considering the Z potential values measured for the nanogel formulations (see Table 1). Indeed, all the formulations present a negative value which ranged between −27 and −41 eV. Instead, as shown in Figure 4B, we reported the intensity profile of nanogels prepared according to the others two procedures and with an HLB = 10. From the inspection of Figure 4B we observe that the top-down methodology results in formation of nanostructures that are slightly smaller (174 ± 82 nm) than those obtained with the W/O emulsion technique, whereas the nanogelling in water methodology provides larger objects (358 ± 186 nm). Moreover, all the nanostructures preserve high stability up to one month.

**Figure 5:**
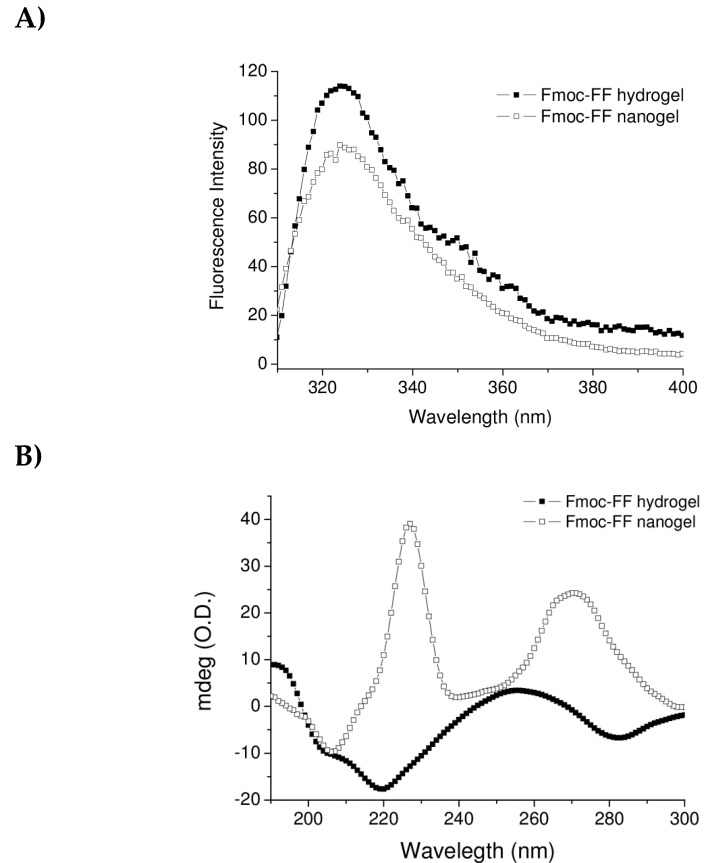
Structural characterization of nanogels prepared by inverse emulsion method. Fluorescence spectrum (**A**) and circular dichroism (CD) spectrum (**B**) of Fmoc-FF nanogel formulation as compared with fluorescence and CD spectra of Fmoc-FF hydrogel.

Aggregation properties and secondary structuration of Fmoc-FF-based nanogels were also characterized by fluorescence and circular dichroism (CD) spectroscopies. Because of the structural role of intermolecular aromatic interactions in gelation, fluorescence spectroscopy has been previously employed to understand the molecular organization of Fmoc-FF building blocks in HG formulations [34,35]. It was observed that the fluorescence emission peak λem = 313 nm) of the fluorenyl moiety in its monomeric form could undergo to a red shift in its aggregated form. The wavelength of the fluorenyl moiety, in its associated form, could provide information on the mutual arrangement occurring in the aggregate. A comparison of the fluorescence spectrum of Fmoc-FF nanogel prepared according to the top-down technique with the spectrum of the macroscopic Fmoc-FF hydrogel is reported in Figure 5A.

As previously reported for other Fmoc-containing aggregates, [34,35], the spectrum of the peptide hydrogel exhibits a maximum at 325 nm, indicating an anti-parallel staking of two fluorenyl moieties. Analogously, it can be noted that the fluorescence spectrum of the nanogel formulation is well superimposable with the spectrum of the hydrogel. This result proves that Fmoc-FF in the nanogel preparation keeps its ability to generate π–π interactions between the fluorenyl moieties, and that the nanosized formulation does not alter the organization at the molecular level.

Further structural information on nanogels was achieved by CD characterization. This spectroscopic methodology has been commonly applied to investigate the secondary structuration of proteins and peptide-based materials [36,37,38]. Indeed, secondary structural types, including α-helix, β-sheet, and random coil, generate distinctive CD spectra. Thus, by comparing the dichroic behaviors of different systems of interest, CD allows one to visualize homologies or differences in secondary structure. The CD spectrum for nanogel formulation (top-down technique) is compared with the spectrum of macroscopic Fmoc-FF hydrogel (black squares) and reported in Figure 5B. The spectra are both characterized by two leading signals. The first signal is located in the 220–230 nm region (221 and 228 nm for Fmoc-FF hydrogel and nanogel, respectively) and it is generally referred to as indicative of β-sheet structuration of the peptide building block in the supramolecular system [39,40]. This signal is negative for Fmoc-FF hydrogel and positive and more intense for nanogel formulation. The dichroic inversion can be attributed to a global different chiral environment in the nanogel as compared with the Fmoc-FF hydrogel. However, this tri-dimensional surrounding does not alter the fundamental β-sheet organization of the Fmoc-FF-based nanogel. Instead, the second signal, which can be considered to be the typical signature of the Fmoc moiety, is a broad band centered at 259 nm for the hydrogel material and it is red shifted at 270 nm for nanogel [25]. The bathochromic effect can be a consequence of a different dielectric constant in the nanogel core covered by surfactants with respect to the macroscopic nude hydrogel. It is worthwhile noting that the two samples also differ in their physical state, and thus the scattering phenomena could also contribute to the red-shifted behavior [41].

### 2.3. Drug Loading and Release

In order to assess the capability of the Fmoc-FF-based nanogels to encapsulate active pharmaceutical ingredients (APIs), nanogels were loaded with doxorubicin (Dox), a well-known water-soluble anticancer drug. Dox belongs to the anthracycline family and works as a DNA intercalating agent and as an inhibitor of topoisomerase II [42], and due to its high cardiotoxicity, Dox can be administered as a liposomal formulation in patients with cardiac compliance. PEGylated liposomal doxorubicin is commercially available as Doxil^®^ (Caelyx^®^ in Europe). FDA approved Doxil, in 1995, for the following: (1) AIDS-related Kaposi’s sarcoma (KS); (2) relapsed ovarian cancer, after platinum-based treatment; (3) metastatic breast cancer with cardiac risk; and (4) multiple myeloma in combination with bortezomib (Velcade^®^). In the latter formulation, Doxil is encapsulated in liposomes using a well-known method based on a sulfate ammonium gradient [43].

Analogously, we formulated Dox-loaded nanogels, according to the top-down methodology, in the absence or in the presence of the sulfate ammonium gradient. The amount of Dox used was chosen in order to obtain a drug loading content (DLC) of 0.250. After the loading, the free drug was removed from nanogels encapsulating doxorubicin using size exclusion chromatography. The size and the zeta potential of the Dox-loaded nanogels were 241.5 nm and −30.2, respectively. The DLC of both of the PBNs was estimated by UV-Vis spectroscopy. Because of the soft nature of the hydrogel disk which was prepared in the ammonium sulfate solution, the resulting encapsulation degree was found to be extremely low (data not shown). On the contrary, in the absence of salt, the measured DLC (0.137) was found to be comparable with that of the not PEGylated liposomal Dox formulation, Myocet (0.127), and slightly lower than that of the PEGylated formulation, Doxil (0.250).

**Figure 6:**
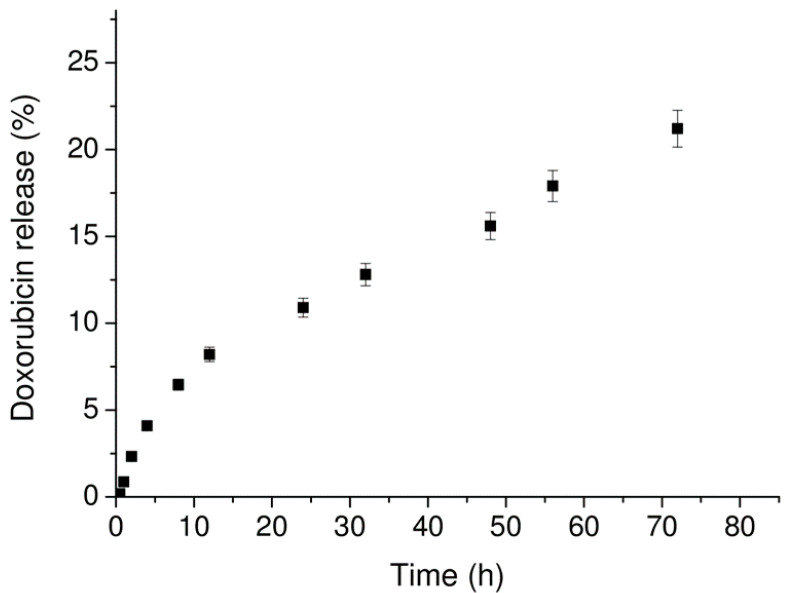
Doxorubicin release profile by Fmoc-FF PBNs. The amount of Dox released was estimated by fluorescence spectroscopy at 590 nm.

The release profile of doxorubicin from nanogels was studied within 72 h, using a dialysis membrane immersed in phosphate buffer at 37 °C. We assumed that the crossing of the free Dox through the dialysis membrane occurred quickly, thus, the overall release of the free drug from the PBNs to the dialysis bag medium could be considered to be rate determining for the process. The amount of Dox released was evaluated by fluorescence spectroscopy, and the releasing profile is reported in Figure 6, as a percentage of released Dox on the total one previously encapsulated in the nanogel. Doxorubicin release (%) for Fmoc-FF nanogel is around 20% after 72 h, with the most part (~50%) released during the first 8–12 h. This value is significantly lower with respect to the release (80% after 55 h) previously observed by Gazit’s group for Fmoc-FF nanogel prepared using E-TPGS surfactant [19].

## 3. Discussion

In the literature, there are only two examples of peptide-based nanogels reported, i.e., the first example concerning the preparation of nanogels using the inverse W/O emulsion technique and the other example concerning a top-down methodology. In this study, we started trying to reproduce nanogels according to the reverse emulsion technique described by Gazit et al., in which the authors used Fmoc-FF peptide as a hydrogelator and E-TPGS, a derivative of the vitamin E, as the stabilizing agent. PEGylated Vitamin E-TPGS was selected due to its remarkable biocompatibility, biodegradability, and low immunogenicity profiles. After their preparation, the authors chose to lyophilize Fmoc-FF nanogels and to resuspend them at the time of use. According to this procedure, we obtained nanoparticles with a mean diameter of ≈250 nm and we traced their stability over the time. The DLS measurements highlighted a significant increase in the size of nanogels (mean diameter around 1000 nm) after the first 24 h from their preparation, thus, indicating that the lyophilization step was indispensable when this formulation procedure was used.

In order to avoid lyophilization, we tried to develop novel and more stable PBN formulations. In this context, we focused our attention on the surfactant stabilizing capacity. Our reasoning was based on the HLB parameter, proposed by Griffin between the late 1940s and early 1950s. To obtain a water-in-oil emulsion, the HLB value of the surfactant should be between three and six, but the E-TPGS has an HLB value of 13.2. It must be said that, in the reverse emulsion method, the two phases are physically and chemically different. Although for the formation of the emulsion it is necessary to obtain stabilization of the aqueous phase in the oily phase, after the extraction of the mineral oil and the rehydration, the system needs to be stabilized in water. For this reason, the first choice was to use an HLB value of 10, which was in the middle of the proposed scale of values. Indeed, surfactants with HLB > 10 are good O/W emulsion stabilizer, whereas surfactants with HLB < 10 are good W/O emulsion stabilizer.

We chose SPAN^®^ 60 and TWEEN^®^ 60 as the surfactants. SPAN^®^ 60 is a hydrophilic neutral surfactant which has an HLB value of 4.7 while TWEEN^®^ 60 is a lipophilic neutral surfactant which has an HLB value of 14.9. By mixing these surfactants in different proportions, it is possible to obtain all the HLB values ranged between 4.7 and 14.9, as described in Table 2. As already mentioned, assuming that an HLB value of 10 could be the most suitable for the stabilization of the formulations, we started to prepare Fmoc-FF nanogels, through the reverse emulsion method, using a mixture of SPAN^®^ 60 and TWEEN^®^ 60 for an HLB = 10. The mean diameter measured for these nanogels, freshly prepared, and after one month in solution at room temperature, was 204 and 224 nm, respectively. The slight increase (around 10%) of the nanoparticles over the time indicates good shelf stability. This result points out that it is possible to avoid the freeze-drying step by choosing an appropriate surfactant. According to the W/O emulsion methodology, we prepared others PBN formulations using different ratios of SPAN^®^ 60 and TWEEN^®^ 60, as reported in Table 1. All the formulations exhibited good stability, even if they showed slightly more uneven profiles at DLS analysis. Surprisingly, we obtained a high stability, also at HLB values comparable or even higher than 13.2, which corresponds to the HLB value of E-TPGS. We suppose this happens because HLB is a parameter that expresses the correlation between the molecular weight of the hydrophilic and the lipophilic parts and provides an indication of the intrinsic tendency of the surfactant to preferentially place itself in the aqueous or oily phase of the emulsion.

However, HLB does not provide information about the stabilizing properties of one surfactant related with another one. Indeed, TWEEN^®^ 60 and E-TPGS have almost comparable HLB values, by presenting a similar number of ethylene glycol groups (polyethylene glycol chains and PEG chains) and a similar length of hydrophobic alkyl chains. Nevertheless, the best polysorbate stabilization capability could be ascribed to the distribution of the PEG chains on different OH groups in TWEEN^®^ 60. This structural feature probably confers conformational freedom to the PEG chain that can orientate themselves towards the aqueous phase, undergoing less folding. Furthermore, it is possible to observe a moderate linear dependence between the nanogel size and the HLB value, since the smaller dimensions are obtained at lower HLB values.

The structural characterization, carried out by fluorescence and CD spectroscopies, highlighted that the inner structure of the Fmoc-FF hydrogel is maintained also in the nanogel formulations. After choosing the best mixture of surfactants, Fmoc-FF nanogels were also prepared according to the other two methods, i.e., the “top-down” and the “nanogelling in water” methods. The top-down method consists of adding the preformed Fmoc-FF hydrogel to an aqueous surfactant solution; whereas the nanogelling in water method is a variant of the inverse emulsion, in which the hydrogel is added to an aqueous solution of stabilizing agents (lacking the mineral oil) before the gelling procedure is completed. The structural characterization highlighted that both methods resulted in obtaining a stable formulation. However, the mean diameter of aggregates prepared by the nanogelling in water method (~360 nm) is two-fold those of the top-down and W/O emulsion methods (204 and 174 nm, respectively). These results suggest that stability is not significantly affected by the preparation method, whereas it seems that the stabilizing agents play a determinant role. The resulting Fmoc-FF-based nanogels were found to be able to encapsulate the model drug doxorubicin with a DLC value comparable to the commercially available liposomal formulations.

## 4. Materials and Methods

Lyophilized Fmoc-FF powder was purchased from Bachem (Bubendorf, Switzerland). TWEEN^®^ 60, SPAN^®^ 60, oil mineral, and all other chemicals were purchased from Sigma-Aldrich, Fluka (Bucks, Switzerland) or LabScan (Stillorgan, Dublin, Ireland) and were used as received unless otherwise stated. All solutions were obtained by weight using doubly distilled water as a solvent. The effective peptide concentrations in solution were spectroscopically determined by UV-Vis measurements on a nanodrop 2000c spectrophotometer (Thermo Fisher Scientific Inc., Wilmington, DE, USA) equipped with a 1.0 cm quartz cuvette (Hellma) using as molar absorptivity (ε) the values of 7800 mol^−1^ L cm^−1^ at 301 nm.

### 4.1. Nanogel Formulations

#### 4.1.1. Water-in-Oil Emulsion Technique

One milliliter of Fmoc-FF hydrogel at a concentration of 1 %wt was prepared attending the solvent switch method. Briefly, 10 mg of Fmoc-FF were solubilized in 100 μL of dimethyl sulfoxide (DMSO), and then rehydrated with 900 μL of sterilized water. The gel formation was added to a solution of surfactants in 9 mL of mineral oil. Surfactants were alternatively E-TPGS (3 × 10^−5^ mol) or a TWEEN^®^ 60/SPAN^®^ 60 mixture (3 × 10^−5^ mol), at different ratios (*w/w*), as reported in Table 2. The resulting suspension was subjected to homogenization at 35,000 min^−1^ for 5 min. Then, the emulsion was stirred at 4 °C, for 3 h, and then the oily phase extracted three times by centrifugation with 6 mL of n-hexane. The product of the extraction was dried under vacuum for 3 h, and then resuspended into 4 mL of sterilized water. This suspension was sonicated using a tip sonicator for 5 min at 9 W.

#### 4.1.2. Top-Down Methodology

One milliliter of Fmoc-FF hydrogel was prepared according to the switch method. The peptide in the DMSO/water mixture was transferred into a silicone mold before the gelification process was completed in order to obtain a gel disk. This disk was added to 4 mL of an aqueous filtered solution of TWEEN^®^ 60/SPAN^®^ 60 at a w/w ratio of 52/48 (3 × 10^−5^ mol) and the resulting suspension was homogenized at 35,000 min^−1^ for 5 min, and then tip sonicated for 5 min at 9 W.

#### 4.1.3. Nanogelling in Water

Fmoc-FF hydrogel 1% wt, prepared in accordance with the solvent switch method, was added into 4 mL of an aqueous filtered solution of TWEEN^®^ 60 and SPAN^®^ 60 surfactants (52/48 *w/w* and 3 × 10^−5^ mol) before the gelling process was completed. The resulting suspension underwent the same procedure previously described consisting of homogenization at 35,000 min^−1^ for 5 min and tip sonication for 5 min at 9 W.

### 4.2. DLS Measurements

Hydrodynamic radii (RH) diffusion coefficients (D) and the zeta potential (ζ) of all the peptide nanogel formulations were measured by dynamic light scattering (DLS) using a Zetasizer Nano ZS (Malvern Instruments, Westborough, MA, USA). Instrumental settings for the measurements were a backscatter detector at 173° in automatic modality, room temperature, and disposable sizing cuvette as cell. The DLS measurements, in triplicate, were carried out on aqueous samples after centrifugation at room temperature at 13,000 rpm, for 5 min. Data of ζ were collected as the average of 20 measurements.

### 4.3. Fluorescence Measurements

Fluorescence spectra of Fmoc-FF-based nanogels and Fmoc-FF-based hydrogels were recorded at room temperature with a spectrofluorophotometer Jasco (Model FP-750, Japan) with the test sample in a quartz cell with 1.0 cm path length. The other settings were as follows: excitation and emission bandwidths = 5 nm, recording speed = 125 nm/min, and excitation wavelength = 280 nm.

### 4.4. Circular Dichroism

Far-UV CD spectra of aqueous solution of nanogels and hydrogels at the same peptide concentration were collected with a Jasco J-810 spectropolarimeter (Japan) equipped with a NesLab RTE111 thermal controller unit using a 0.1 mm quartz cell at 25 °C. The spectra of samples were recorded from 280 to 190 nm. Other experimental settings were as follows: scan speed = 10 nm/min, sensitivity = 50 mdeg, time constant = 16 s, and bandwidth = 1 nm. Each spectrum was obtained by averaging three scans and corrected for the blank.

### 4.5. Doxorubicin Loading

Dox-loaded nanogels were prepared according to the top-down methodology previously described, with or without the sulfate ammonium gradient. In the classical top-down methodology, a disk of Fmoc-FF hydrogel loaded with Dox was prepared as previously reported, adding the stock solution of Fmoc-FF in DMSO (100 mg/mL) to 900 μL of an aqueous solution of Dox (0.0141 mol L^−1^) in order to reach a drug weight/lipid weight ratio of 0.250. The hydrogel disk was smashed into 4 mL of an aqueous solution of TWEEN^®^ 60/SPAN^®^ 60 by homogenization and sonication. The other Dox-loaded nanogels were prepared using the top-down approach, modified according to the well-assessed ammonium sulfate gradient method [43]. Briefly, empty Fmoc-FF nanogel (HLB = 10) were prepared, as previously described, in a 250 mM sulfate ammonium solution (pH = 5.5). The nanogel solution was eluted on a Sephadex G-50 column pre-equilibrated with HEPES buffer (10 mM) at pH 7.4, then 57.4 μL of doxorubicin from an aqueous stock solution (1.18 × 10^−2^ mol/L) were added to 500 μL of nanogel in order to reach a drug weight/lipid weight ratio of 0.250. The suspension was stirred alternatively for 30 min or overnight at room temperature. For both the preparation, unloaded Dox was removed from the nanogel solution by gel filtration on a pre-packed column Sephadex G-50. The Dox concentration was determined by UV-Vis spectroscopy using calibration curves obtained by measuring absorbance at λ = 480 nm. The drug loading content (DLC, defined as the weight ratio of encapsulated Doxo versus the amphiphilic molecules forming nanogels) was quantified by subtraction of the amount of removed Dox from the total amount of loaded Dox.

### 4.6. Drug Release

The in vitro Dox release from nanogels was measured using a dialysis method [44]. Briefly, 1.0 mL of nanogels loaded with Dox were prepared as previously described and immediately transferred into a dialysis bag (MW cut-off = 3500 Da). Then, it was placed into 20 mL of phosphate buffer and incubated under stirring for 72 h, at 37 °C. Then, 2 mL of the dialyzed solution were replaced with an equal amount of fresh solution at different time points. The extent of drug release was estimated by fluorescence spectroscopy as a percentage of the ratio between the amount of released drug and of the total drug previously loaded into the nanogel. The amount of released Dox was evaluated using a titration curve. For the fluorescence measurements, the excitation wavelength was settled at 480 nm, and the emission spectrum recorded between 490 and 700 nm.

## 5. Conclusions

In recent years, nanogels have been identified as interesting platforms for the delivery of APIs (such as drugs, contrast agents, nucleic acids, and so on). Although there is a large number of studies on polymer-based nanogels, only a few studies have been reported on peptide-based nanogels until now. This is probably related to the difficulty associated with obtaining a stable form indispensable for their commercialization and their use in the pharmaceutical field.

We prepared peptide nanogel formulations based on the well-known hydrogelator Fmoc-FF using three different methods. In these preparations we evaluated the effect of the HLB value of the stabilizing agent used to coat the hydrogel nanoparticles. We proposed a modification of the inverse emulsion technique and the top-down methodology mainly by changing the surfactants. Indeed, we used a mixture of TWEEN^®^ 60 and SPAN^®^ 60 which were able to guarantee good stability over time, after storage in water at room temperature. In this way, the freeze-drying step could be avoided.

Furthermore, we proposed a new formulation method for the preparation of Fmoc-FF nanogels, named nanogelification in water. Nanoparticles obtained by top-down and W/O emulsion have a diameter of about 200 nm, which is desirable for any clinical applications. However, it is worthwhile noting that the top-down methodology exhibits several advantages with respect to the W/O emulsion method. First, the top-down method avoids the use of mineral oil during the preparation. Consequently, the extraction of the nanogel solution with organic solvents such as n-hexane can also be avoided. Moreover, the top-down method requires a minor number of steps during the preparation. The easy procedure, conjointly with the high biocompatibility, are useful characteristics in the perspective to optimize and simplify their industrial fabrication. Moreover, this method is also compatible with procedures to encapsulate anticancer drugs. Even if there is a long way to carry nanogel from bench to bedside for drug delivery applications [45], the use of peptide-based nanogels could contribute to shortening the development phases and the results reported here give a good perspective for the use of PBNs as a new drug delivery system.

## Figures and Tables

**Table 1:** Structural characterization (mean diameter, polydispersity index (PDI), and zeta potential) of Fmoc-FF nanogels prepared according to the three methods.

Method	HLB	Mean Diameter (nm) ± S.D.	PDI	Mean Diameter (nm) ± S.D. After 30 gg	ζ mV± S.D.
W/O emulsion	4.7	163 ± 87	0.180	189 ± 89	−40.2 ± 0.3
W/O emulsion	6	205 ± 85	0.174	241 ± 91	−40.9 ± 1.5
W/O emulsion	8	217 ± 93	0.201	249 ± 102	−30.4 ± 0.9
W/O emulsion	10	204 ± 115	0.192	224 ± 109	−27.4 ± 1.0
W/O emulsion	12 ^[a]^	*d*_1_ = 161± 42 *d*_2_ = 721 ± 230	0.168 0.264	*d*_1_*=* 243 ± 117 *d*_2_ = 1844 ± 560	−29.5 ± 1.0
W/O emulsion	14.9	242 ± 100	0.208	332± 183	−27.2 ± 1.0
Top-down	10	174 ± 82	0.176	202 ± 97	−24.0 ± 0.1
Nanogelling in water	10	*d*_1_*=* 61 ± 18 *d*_2_ = 358 ± 186	0.132 0.210	*D*_1_ = 98 ± 43 *d*_2_ = 407 ± 235	−16.6 ±0.6
Top-down/Dox	10 ^[a]^	242 ± 102	0.190	----	−30.2 ± 0.3

^[a]^ Two populations of aggregates (indicated as *d*_1_ and *d*_2_) were found for the W/O emulsion HLB 12 and for nanogelling in water HLB 10.

**Table 2:** Hydrophilic–lipophilic balance (HLB) values obtained combining different percentages of SPAN^®^ 60 and TWEEN^®^ 60.

Emulsifier Blend		HLB
SPAN^®^ 60	TWEEN^®^ 60	
100%	0%	4.7
87%	13%	6
68%	32%	8
48%	52%	10
28%	72%	12
6%	94%	14
0%	100%	14.9
